# Cases Illustrating the History of a Peculiar Local Disease, Apparently Produced by the Application of a Poisonous Matter Contained in Offal

**Published:** 1827-04

**Authors:** B. C. Brodie


### POISONOUS MATTER IN OFFAL.

*Cases illustrating the History of a peculiar Local Disease, apparently produced by the Application of a Poisonous Matter contained in Offal.* By B. C. Brodie, F.R.S. &c.

16, *Saville-row; March* 18, 1827.

Dear Sir,—I send you an account of some of the cases which I lately mentioned to you, in which a remarkable train of local symptoms followed the handling offal. In the first of these cases, the disease was allowed to take its own course, and it subsided spontaneously in about six weeks. In the two other cases, medical treatment was employed with apparent advantage. You may recollect that I remarked in conversation, that, as such cases are not very uncommon, I had no doubt that the disease had been observed by others, although, as far as I knew, it was not described by authors. In confirmation of this opinion, I have since been informed by my friend Mr. Travers, that he has seen several persons affected in a similar manner, and from the same cause, whose symptoms appeared to be relieved by the exhibition of the Pilula Hydrargyri.

I am, dear Sir, your obedient servant,

*Dr. Macleod.* B. C. BRODIE.

Case I.—A. B., a healthy young man, on the 8th of September, 1820, while engaged in feeding dogs with sheep's offal, cut the forefinger of his left hand near the tip. The wound was slight, and healed in the course of two or three days; but, as soon as the healing was completed, the end of the finger was observed to be inflamed and swollen as far as the second joint: from thence the inflammation slowly extended over the first phalanx of the forefinger; next up the outside of the hand, as high as the wrist; then downward over the middle finger; again upwards on the palm of the hand to the wrist; and again downward over the whole of the ring finger, and the first phalanx of the little finger. The inflammation was marked by a deep redness of the skin, with slight tumefaction and much tenderness. The tumefaction and tenderness were greatest in the situation of the joints of the fingers, so as to occasion much difficulty of moving them. The margin of the inflammation was less defined than that of erysipelas, but more so than that of common phlegmonous inflammation. It was observed that the redness occupied only a small portion of the hand and fingers at one time, leaving the part previously affected as it spread to a new surface; but that the swelling and tenderness remained a considerable time after the redness had disappeared, especially in the neighbourhood of the joints. The general health was unaffected, and there was no enlargement of the lymphatic glands in the axilla, or above the inner condyle of the arm.

On the 14th of October, there were only some slight remains of the disease in the little finger; and in the course of a short time afterwards the symptoms had entirely vanished.

Case II.—J. W., the cook at an hotel, on the 10th of October, 1821, scratched his finger with the extremity of a rib, while taking out the viscera of a hare. The scratch was on the inside of the second phalanx of the forefinger. In the course of two or three days, he observed an appearance of inflammation in the situation of the hurt, marked by a slight degree of redness and swelling, and by tenderness. The inflammation extended up the forefinger to the back of the hand, and from thence it passed downwards over the middle finger, subsiding in one part as it attacked another.

On the 31st of October, when I first saw the patient, there was inflammation of the skin covering the first phalanx of the middle finger, and also of that on the back of the hand, nearly as high as the wrist. The inflamed parts were of a crimson red colour, and sufficiently painful and tender to prevent the hand being used. The general health was not affected. Leeches had been applied several times before I was consulted, without benefit. I prescribed some local applications, which, however, did not seem to have any influence over the disease.

On the 8th of November, therefore, I recommended that one-eighth of a grain of the oxymuriate of mercury should be taken twice daily, in the form of a pill.

On the 27th of November, the redness and swelling were much abated, but there was considerable pain in the hand, extending up the forefinger. In addition to the oxymuriate of mercury, ℨj. of the powder of sarsaparilla was directed to be taken three times daily.

December 4th.—The redness and swelling had nearly disappeared; but the patient complained of what he called a *numbed* pain, extending up the forearm and arm as high as the shoulder.

23d.—The pain in the arm and forearm not being relieved, a blister was applied to the arm. No alteration was made in the other part of the treatment.

31st.—The pain in the arm was much relieved, but that in the forearm continued unabated. A blister was applied to the anterior part of the forearm. The internal remedies were discontinued.

January 12th, 1822.—The pains in the limb were much diminished. It was directed that the arm and forearm should be rubbed with a stimulating liniment two or three times daily.

26th.—There were no remains of the disease.

Case III.—A middle-aged woman, on the 14th of October, 1826, pricked the middle finger of the left hand with a splinter of bone, in cleaning the inside of a hare, of which the viscera had been previously removed. The prick was at the posterior part, near the nail. On that day she suffered no inconvenience, but on the following day, October 15th, there was a slight inflammation near the puncture. During the following week the inflammation increased, and it was attended with a good deal of pain, so as to prevent sleep at night.

On Monday the 23d of October, when I first saw the patient, the whole of the middle finger was inflamed and swollen, but the inflammation was greatest in the second and third phalanx; and here the skin was red, tense, and shining. She complained of a tingling and throbbing pain, which prevented sleep; but there was little or no disturbance of the general system, beyond what sleepless nights might have occasioned. I prescribed one-eighth of a grain of the oxymuriate of mercury to be taken twice daily, and the application of a poultice.

On the 24th of October, the inflammation at the end of the finger had abated, but it had extended to the back of the hand.

25th.—The inflammation had nearly subsided in the middle finger, but it had crossed over the back of the hand to the ring finger; and on the following day, October 26th, it had extended to the forefinger also.

On the 28th of October, the middle finger was still somewhat swollen, but it exhibited no other marks of inflammation. There was, however, considerable inflammation, marked by pain, tenderness, and redness of the skin on the back of the hand, and extending from thence to the back part of the forefinger and ring finger. She described the pain as attended with a sense of tingling and throbbing, less severe than that which she endured formerly, and not sufficient to interrupt her sleep. She was directed to continue the use of the oxymuriate.

November 2d.—The inflammation had extended no further, and was much abated in the parts formerly affected; and from this time she continued rapidly to mend.

15th.—No marks of the disease were left, and she was directed to discontinue taking the medicine.

